# A Study of Candidate Genes Associated with Suicide Attempts in the Kazakh Population in Astana, Kazakhstan

**DOI:** 10.3390/ijms27052294

**Published:** 2026-02-28

**Authors:** Roza Tatayeva, Aruzhan Tussupova, Akmaral Nursafina, Elena Zholdybayeva, Zhannat Bazarbayeva, Olga Fedorenko, Zhibek Sembaeva, Aigul Tulembaeva, Saule Sarkulova, Botagoz Karimbayeva

**Affiliations:** 1 Department of General Biology and Genomics, L.N. Gumilyov Eurasian National University, Astana 010000, Kazakhstan; aruzhan_1803@mail.ru (A.T.); bazarbayeva@inbox.ru (Z.B.); zhibek.sembayeva@yandex.ru (Z.S.); 2 Department of Technology and Standardization, K. Kulazhanov Kazakh University of Technology and Business, Astana 010000, Kazakhstan; 3 National Scientific Shared Laboratory of Biotechnology, National Center for Biotechnology, Astana 010000, Kazakhstan; zholdybayeva@biocenter.kz; 4 Mental Health Research Institute, Tomsk National Research Medical Center, Russian Academy of Sciences, Tomsk 643000, Russia; f_o_y@mail.ru; 5 Ministry of Defense of the Republic of Kazakhstan, Astana 010000, Kazakhstan; tulembaeva@mail.ru; 6 Institution of NJSC “Astana Medical University”, Astana 010000, Kazakhstan; saule_sark@mail.ru; 7 Department of Social and Humanitarian Disciplines, Kazakh National University of Arts, Astana 010000, Kazakhstan; karimbayeva@mail.ru

**Keywords:** suicidal behavior, suicide attempts, genetic polymorphisms, *HTR2A*, *SKA2*, serotonergic system, hypothalamic–pituitary–adrenal axis, stress reactivity, sex-specific effects, candidate genes, Kazakh population, molecular psychiatry

## Abstract

Suicidal behavior is a multifactorial and highly heritable phenotype; however, data concerning its genetic determinants in disparate ethnic groups remain limited. Genes implicated in serotonergic neurotransmission and stress response regulation are regarded as primary candidates for elucidating biological vulnerability to suicide. The objective of this study is to investigate the relationship between suicide attempts and candidate gene polymorphisms in an ethnically homogeneous Kazakh population from Astana, Kazakhstan. The study’s sample population comprised 126 patients with a documented history of suicide attempts and 120 age- and gender-matched controls without a history of suicidal behavior. A comprehensive genotyping analysis was conducted, encompassing polymorphisms in genes associated with serotonergic signaling, stress response, and neuroplasticity (*TPH1*, *TPH2*, *HTR2A*, *MAOA*, *SLC6A4*, *ANKK1*, *BDNF*, *COMT*, *CXCL8*, *SKA2*, and *FKBP5*). The associations were assessed across several genetic models, using odds ratios with 95% confidence intervals. A substantial correlation was identified between the *HTR2A rs6311* polymorphism and suicide attempts. The CC genotype exhibited a protective effect (*p* = 1.36 × 10^−5^), while the TT genotype was associated with an elevated risk (OR = 3.16; 95% CI: 1.72–5.81). The association remained robust after stratification by sex, with an even stronger effect observed in women (OR = 4.70; 95% CI: 2.08–10.64). A nominal sex-specific association was observed for the *SKA2 rs7208505* variant, suggesting a potential role in stress-response mechanisms in women; however, this association was no longer statistically significant after adjustment for multiple comparisons. These results identify *HTR2A rs6311* as a potential genetic marker of suicide risk in the Kazakh population and support the involvement of serotonergic receptor regulation in the biological mechanisms underlying suicidal behavior. The results underscore the significance of sex-specific genetic influences, thereby enhancing our understanding of the polygenic underpinnings of suicidality.

## 1. Introduction

Suicidal behavior is a multifactorial phenomenon caused by the complex interaction of biological, psychological, social, and cultural factors. In recent decades, it has been identified as one of the primary causes of premature mortality on a global scale and a substantial public health concern [1]. According to a report by the World Health Organization (WHO), one individual dies by suicide every 40 s, amounting to more than 800,000 deaths annually. Furthermore, for every death by suicide, there are more than 20 suicide attempts [2]. According to estimates derived from twin and family studies, the heritability of suicidal behavior ranges from 30 to 50%, suggesting a substantial contribution of genetic factors in the development of individual vulnerability [3,4,5]. The prevailing hypothesis posits that genetic predisposition manifests through its influence on intermediate neurobiological phenotypes, including emotion regulation, impulsivity, stress reactivity, and cognitive control [6]. An analysis of epidemiological data indicates that Kazakhstan is among the countries with relatively high suicide mortality rates. The prevalence of suicidal behavior demonstrates pronounced age specificity, with a peak observed among individuals aged 15–19 years [7]. Among the socioeconomic factors examined, unemployment has been identified as a particularly salient predictor of elevated suicide rates, particularly among male populations [8]. Despite this, the biological mechanisms of suicidal behavior in Central Asian populations remain poorly understood, while most genetic studies have been conducted primarily in European and East Asian samples [9,10,11,12]. This underscores the necessity for population-specific studies that are designed to identify the molecular determinants of suicide risk.

Current neurobiological models of suicidal behavior posit the involvement of several interconnected signaling systems, including serotonergic and dopaminergic neurotransmission, hypothalamic–pituitary–adrenal (HPA) axis regulation, neuroplasticity, and neuroinflammation [6,13,14,15]. Dysfunction of these systems can disrupt adaptive stress-response mechanisms, increase emotional reactivity, and lead to the development of destructive behavioral strategies.

Among the biological systems involved in the pathogenesis of suicidal behavior, serotonergic neurotransmission plays a key role. Serotonin (5-hydroxytryptamine, 5-HT) is a neurotransmitter that plays a role in the regulation of mood, anxiety, aggression, impulsivity, and sleep. It is considered a central neurotransmitter associated with suicidality [16]. The functioning of the serotonin system is ensured by the coordinated action of genes encoding enzymes of synthesis (*TPH1*, *TPH2*), transport (*SLC6A4*), and metabolism (*MAOA*) of serotonin, as well as its receptors (*HTR1A*, *HTR2A*, *HTR2C*) [17]. Genetic polymorphisms of these genes have been demonstrated to result in a decrease in the availability of serotonin [18], disruption of signal transmission through serotonin receptors, and changes in stress reactivity. These alterations contribute to the formation of pathological behavioral patterns, including suicidal tendencies [19,20,21].

In the context of suicidal behavior, particular attention has been given to the *HTR2A* gene, which encodes the 5-HT2A receptor. This receptor is widely expressed in the prefrontal cortex, neocortex, and hippocampus—structures that are critically involved in regulating emotion, cognitive control, and behavior [22]. Among the polymorphisms with potential functional significance are *rs6311* (*T102C*) and *rs6313* (*A1438G*), both of which have the capacity to affect receptor expression and alter sensitivity to serotonin [23]. However, the existing data on the association of these polymorphisms with suicidal behavior remain contradictory and have not previously been studied in the Kazakh population [24].

In addition to receptor mechanisms, the genes involved in serotonin biosynthesis may play an important role. The *TPH1* and *TPH2* genes, which encode isoforms of tryptophan hydroxylase—a pivotal enzyme in serotonin biosynthesis—are also regarded as significant candidates in the context of suicidal behavior research [25,26]. Polymorphic variants of *TPH1*, including *A218C*, have been associated with changes in serotonin metabolism and an increased risk of mental disorders and suicidal tendencies [27,28]. Additionally, studies have identified tryptophan metabolism disturbances in patients with depressive disorders and suicidal tendencies [29]. Functional polymorphisms in *TPH2* have been described, including *rs4570625*, which is associated with variability in the stress response and affective states. However, the research results remain controversial [30,31].

The *SLC6A4* gene, which encodes the serotonin transporter, plays a pivotal role in regulating the synaptic availability of serotonin [32,33]. The *5-HTTLPR* polymorphism has been linked to heightened amygdala reactivity to emotional stimuli, anxiety, and an elevated risk of addiction, particularly in conjunction with depressive and anxiety disorders [34,35]. However, its role in suicidal behavior remains a subject of debate.

Genes implicated in the regulation of monoaminergic neurotransmission, including *MAOA*, *ANKK1*, and *COMT*, are regarded as significant candidates for research on suicidal behavior. This is due to their involvement in catecholamine metabolism, impulsivity regulation, and behavioral control. The *MAOA* gene, which is located on the X chromosome, is responsible for encoding the enzyme monoamine oxidase A (MAOA). This enzyme plays a crucial role in the degradation of neurotransmitters such as serotonin, dopamine, and norepinephrine. The *MAOA* gene influences the regulation of mood, motivation, and cognitive function in humans [36,37]. Variations in the regulatory region of the gene, including *VNTR* polymorphisms, have been demonstrated to modulate the level of enzyme expression and monoamine concentrations in the brain [38,39]. Although low-activity alleles have previously been associated with aggression and antisocial behavior, data on the association of *MAOA* with suicidality do not allow for definitive conclusions [40,41,42,43,44,45].

The *ANKK1* gene encodes a protein containing ankyrin repeats and a kinase domain and is considered to be functionally linked to the dopaminergic system [46]. Of particular interest is the *Taq1A* polymorphism (*rs1800497*), which was previously associated with the dopamine receptor gene *DRD2*. However, subsequent research has attributed this polymorphism to *ANKK1*. It has been posited that the impact of this variant on the risk of mental and behavioral disorders, including addictive and suicidal behavior, may be mediated by alterations in dopamine signaling pathways [47,48,49]. A substantial body of research has demonstrated that the TT *rs1800497* genotype functions as an independent risk factor for suicide attempts, both in the overall sample and following gender-based stratification [50]. This finding supports its inclusion in the present study.

The *COMT* gene, which is responsible for encoding the catechol-O-methyltransferase enzyme, plays a pivotal role in the metabolic process of catecholamines, such as dopamine, norepinephrine, and epinephrine [51,52]. The functional polymorphism *rs4680* (*Val158Met*) exerts a substantial influence on the catalytic activity of the enzyme. Carriage of the Met allele is associated with reduced *COMT* activity and elevated dopamine levels in the prefrontal cortex, which may, in turn, affect cognitive control, emotion regulation, and impulsivity [53,54]. It has been posited that these associated changes may impact cognitive control and emotional resilience. However, the results of association studies demonstrate a pronounced population dependence [55].

The regulation of the stress response is also considered a critical component of biological vulnerability. The *FKBP5* and *SKA2* genes are considered to be pivotal elements in the regulation of the hypothalamic–pituitary–adrenal axis (HPA) and the stress response. The dysfunction of these genes has been demonstrated to contribute to the development of suicidal behavior. The *FKBP5* gene, which is associated with the regulation of glucocorticoid receptor sensitivity and the effectiveness of cortisol negative feedback, is a subject of particular interest [56]. Genetic variations in the *FKBP5* gene have been associated with alterations in gene expression and epigenetic modifications, particularly in conditions of early psychosocial stress. These variations have been linked to increased stress reactivity, emotional dysregulation, and reduced stress adaptation [57,58,59,60]. It has been posited that *FKBP5* dysregulation may serve as a mediator for the association between chronic stress, HPA axis dysfunction, and alterations in neuronal plasticity within corticolimbic brain structures, which could potentially elevate the risk of suicidal behavior [61,62]. However, data from association studies of *FKBP5* remain limited and largely contradictory, particularly across ethnic populations.

The *SKA2* gene (*FAM33A*) is known to encode a protein that is associated with the spindle and kinetochore [63]. Decreased expression of the *SKA2* protein has been associated with the development of depressive disorders and suicidal behavior [64]. In the context of neuropsychiatric disorders, *SKA2* is of particular interest due to its role in regulating the stress response. The SKA2 protein is involved in the translocation of the glucocorticoid receptor into the cell nucleus, thereby modulating cortisol sensitivity and the effectiveness of HPA axis negative feedback [65]. As demonstrated in the studies conducted by Guintivano et al. (2014) and Niculescu et al. (2015), there was a decrease in *SKA2* expression and an alteration in epigenetic regulation in individuals who died by suicide [66,67]. These studies also demonstrated the high prognostic value of this gene as a peripheral biomarker of suicidality in blood samples [66,67].

Genes that play a role in the regulation of neuroplasticity and the inflammatory response are considered important components of biological vulnerability to suicidal behavior. *BDNF* (brain-derived neurotrophic factor) plays a pivotal role in the processes of proliferation, differentiation, and survival of neurons, as well as in maintaining synaptic plasticity. The highest *BDNF* expression has been observed in the hippocampus and cerebral cortex—structures involved in the regulation of cognitive and emotional functions [68]. The functional polymorphism Val66Met (*rs6265*) has been demonstrated to influence intracellular transport and activity-dependent secretion of *BDNF.* This variant has been previously linked to depressive disorders, suicide attempts, cognitive impairment, and decreased neuroplasticity, particularly in conditions of chronic stress [69,70].

Furthermore, neuroinflammation is regarded as a promising field in the search for biomarkers of suicidal behavior. Interleukin-8 (IL-8), which is encoded by the gene identified as *CXC8*, belongs to the CXC-chemokine family. It plays a pivotal role in the inflammatory response, regulating immune cell migration and activation through interactions with specific receptors and glycosaminoglycans [71,72]. The *rs1126647* polymorphism of the *CXCL8* gene has previously been associated with inflammatory diseases and a number of mental disorders, suggesting it as a potential genetic marker of inflammation-mediated psychopathological phenotypes [73]. However, genetic studies of the chemokine *CCL8* in the context of suicidal behavior remain limited, particularly in non-European populations, highlighting the need for further research considering the interactions between inflammatory, neurotransmitter, and stress-regulatory mechanisms.

Consequently, suicidal behavior is influenced by a multifaceted interplay of serotonergic, monoaminergic, stress-regulatory, neuroplastic, and inflammatory mechanisms. Despite the substantial body of data obtained primarily in European populations, the role of genetic factors in suicidal behavior in the Kazakh population remains to be elucidated. The novelty of this study lies in its comprehensive assessment of polymorphisms in the *genes TPH1* (*rs211105*, *rs7933505*), *TPH2* (*rs4570625*, *rs4641528*, *rs13886494*), *HTR2A* (*rs6313*, *rs6311*), *MAOA* (*rs5906957*), *SLC6A4* (*rs6355*), *ANKK1* (*rs1800497*), *BDNF* (*rs6265*), *COMT* (*rs4680*), *CXCL8* (*rs1126647*), *SKA2* (*rs7208505*), and *SLC6A4* (*rs6355*) in representatives of the Kazakh population who attempted suicide, taking into account possible sex differences. The objective of the present study was to evaluate the associations between suicide attempts and candidate gene polymorphisms, with the aim of deepening our understanding of the molecular genetic mechanisms of suicidal behavior and identifying potential genetic risk markers.

## 2. Results

### 2.1. Characteristics of the Study Participants

The present study comprised 246 participants. Of the 126 patients with documented suicide attempts, 38 (30%) were male, and 88 (70%) were female, yielding a female-to-male ratio of 1:0.43. The mean age ± standard deviation (SD) of suicide attempt cases was 36.23 ± 15.9 years. The mean values for men and women were 41.21 ± 14.86 and 33.85 ± 15.87, respectively (see Table 1). The 18–29 age group was the most prevalent, accounting for 47.6% of patients, 80% of whom were female.

The control group consisted of individuals who were matched for age, gender, and ethnicity (Kazakhs). The mean age ± SD of the control group was 33 ± 12.74 years. Of the 120 subjects, 37 (30.8%) were male, and 83 (69.2%) were female. The mean age was 34.48 ± 15.25 years for men and 32.55 ± 11.55 years for women (see Table 1). A subsequent analysis of the gender distribution between the study and control groups revealed no significant differences (*p* > 0.05).

The study found no statistically significant differences between the groups in terms of age or gender, indicating adequate comparability between the samples. Marital status also did not differ significantly between the groups. However, substantial discrepancies were observed in educational level (*p* = 0.01) and occupational status (*p* < 0.001). Patients who had attempted suicide were more likely to be unemployed, while the control group was more likely to be employed. Moreover, a lower proportion of patients had less than a college degree compared to the control group.

The clinical diagnoses were assessed exclusively within the patient group, with adjustment disorder being the most prevalent condition, followed by alcohol use disorder and major depressive episodes without psychotic symptoms.

### 2.2. Results of the Distribution of Genotype Frequencies by Loci

The overall genotype and allele distribution for the analyzed SNPs is presented in Table 2 for descriptive and quality assurance purposes. For greater analytical transparency, allele frequencies stratified by case–control status are additionally reported in Supplementary Table S1, enabling a more detailed evaluation of between-group differences. The analysis of associations with suicide attempts was conducted taking into account age categories; however, no statistically significant differences in the genotype distribution between patients and controls in age subgroups were found (*p* > 0.05).

For the *rs211105* polymorphism of the *TPH1* gene, the genotype distribution complied with the Hardy–Weinberg equilibrium, and the minor allele frequency was 24.2%. *rs7933505* polymorphism of the *TPH1* gene demonstrated a significant deviation from the Hardy–Weinberg equilibrium and was excluded from further analysis. The *HTR2A* gene polymorphisms (*rs6313* and *rs6311*) were in population equilibrium; the minor allele frequency for *rs6311* was 48.6%. For the *TPH2* gene, the *rs4641528* polymorphism was in Hardy–Weinberg equilibrium, whereas the *rs4570625* and *rs1386494* polymorphisms demonstrated significant deviations from population equilibrium conditions (*p* < 2.2 × 10^−16^) and were excluded from further analysis.

The *rs5906957* polymorphism in the *MAOA* gene, located on the X chromosome, was analyzed in light of the inheritance characteristics of X-linked genes. The *MAOA* gene is located on the X chromosome; because males are hemizygous, the standard test of Hardy–Weinberg equilibrium in a combined sample of males and females is not valid and was not used. The *ANKK1* (*rs1800497*), *COMT* (*rs4680*), *SKA2* (*rs7208505*), and *FKBP5* (*rs3800373*) gene polymorphisms met population equilibrium conditions, and the minor allele frequencies were within the expected ranges for the study population.

The *BDNF rs6265* and *CXCL8 rs1126647* polymorphisms were excluded from further analysis due to significant deviations from Hardy–Weinberg equilibrium (*p* < 2.2 × 10^−16^ and *p* < 1.30 × 10^−7^, respectively). The *SLC6A4 rs6355* polymorphism had an extremely low minor allele frequency and no carriers of the homozygous minor genotype, which significantly limited the statistical power of this SNP.

### 2.3. Results of Association Analysis of Genetic Polymorphisms

As a result of the search for associations, a statistically significant association was revealed between the *rs6311* polymorphism of the *HTR2A* gene and the presence of suicide attempts in the total sample (Table 3). In the additive model, the homozygous CC genotype showed a significant protective effect and was observed at a lower frequency among patients with suicide attempts than in the control group (*p* = 1.36 × 10^−5^). Conversely, the presence of the TT genotype was associated with a more than threefold increase in the risk of suicidal behavior. The results remained statistically significant when analyzed in dominant and recessive inheritance models. In the dominant model, the presence of at least one T allele (CT + TT) was correlated with a diminished frequency of the CC genotype in the suicidal group relative to the control group (*p* = 0.0001). In contrast, the recessive model demonstrated that individuals carrying the CC/CT genotypes exhibited a protective effect, while those with the homozygous TT genotype exhibited a significantly elevated risk of suicide attempts (*p* = 0.0002).

These results indicate a stable and reproducible association between the rs6311 polymorphism of the *HTR2A* gene and suicidal behavior in the general population. For the remaining single-nucleotide polymorphisms that were the focus of this study, no statistically significant associations with suicide attempts were identified in the overall sample, regardless of the specific inheritance model (i.e., additive, dominant, or recessive).

The *rs211105* polymorphism of the *TPH1* gene exhibited no disparities in genotype distribution between patients with suicide attempts and the control group across any inheritance model; odds ratios approached one, and confidence intervals encompassed 1. Correspondingly, no statistically significant associations with suicidal behavior were identified for the *rs6313* polymorphism of the *HTR2A* gene. For the *rs4641528* polymorphism of the *TPH2* gene, genotype frequencies did not differ significantly across the additive, dominant, and recessive models between the study and control groups, despite a tendency toward a lower frequency of the CC genotype among patients with suicide attempts.

The *rs6355* polymorphism of the *SLC6A4* gene was characterized by an extremely low frequency of the minor allele and the absence of carriers of the homozygous minor genotype. Due to the presence of zero values in the cells of the contingency table, the standard chi-squared test was not employed. Instead, the genotype distributions between groups were compared using Fisher’s exact test. No statistically significant differences were found between patients with suicide attempts and the control group (*p* > 0.05). For the *rs1800497* polymorphism of the *ANKK1* gene, the distribution of genotypes between groups was comparable, and the OR and 95% CI values did not indicate an association with suicidal behavior in any of the analyzed genetic models.

Similarly, the *rs4680* polymorphism of the *COMT* gene did not demonstrate statistically significant associations with suicide attempts in additive, dominant, and recessive models, despite a trend toward a higher frequency of the AA genotype among patients.

For the *rs7208505* polymorphism in the *SKA2* gene in the general population, differences in genotype distribution between the study and control groups were not statistically significant.

The *rs3800373* polymorphism of the *FKBP5* gene also showed no significant differences in genotype frequencies between patients and controls across any inheritance model, with confidence intervals for all OR estimates that included 1.

Following the stratification of the data by sex, a series of statistically significant associations were identified that were specific to either sex for the *rs6311 HTR2A* gene polymorphism and the *rs7208505 SKA2* gene polymorphism in women (see Table 4 for details).

For the *rs6311 HTR2A* gene polymorphism, the association with suicide attempts in women was consistent across all inheritance models. In the additive model, the homozygous CC genotype exhibited a significant protective effect and was less prevalent among women with suicide attempts compared to the control group (OR = 0.29; 95% CI: 0.14–0.61; χ^2^ = 20.14; *p* = 4.24 × 10^−5^). Conversely, the homozygous TT genotype was associated with a significantly elevated risk of suicidal behavior (OR = 4.70; 95% CI: 2.08–10.64). In the dominant model, the presence of the CC genotype was associated with a significantly reduced risk of suicide attempts (OR = 0.29; 95% CI: 0.14–0.61; χ^2^ = 37.72; *p* = 8.16 × 10^−10^). In the recessive model, the CC/CT genotype was associated with a significant protective effect (OR = 0.21; 95% CI: 0.09–0.48; χ^2^ = 13.89; *p* = 0.0001), while the TT genotype was associated with a multiple-fold increase in the risk of suicidal behavior.

In an additive analysis of the *rs7208505* polymorphism of the *SKA2* gene, the presence of the AA genotype was associated with an elevated risk of suicidal behavior in women (OR = 2.34; 95% CI: 1.15–4.78), exhibiting borderline statistical significance (*p* = 0.05). Conversely, the AG and GG genotypes did not exhibit independent effects. In the dominant model, no statistically significant differences were found between the groups (*p* = 0.6977). In the recessive model, a pronounced protective effect of the G allele was revealed: the AG + GG genotype combination was associated with a reduced risk of suicide attempts in women (OR = 0.43; 95% CI: 0.21–0.87; *p* = 0.0275), while the homozygous AA genotype remained a risk factor. However, after adjustment for multiple comparisons using the Bonferroni correction, these associations did not reach statistical significance, and the results obtained should be considered nominal.

For the remaining polymorphisms under study, including *TPH1* (*rs211105*), *HTR2A* (*rs6313*), *TPH2* (*rs4641528*), *ANKK1* (*rs1800497*), *COMT* (*rs4680*), and *FKBP5* (*rs3800373*), no statistically significant associations with suicide attempts were identified in the female subgroup in any of the additive, dominant, or recessive inheritance models (*p* > 0.05) (Table 5). The association analysis of the *MAOA rs5906957* polymorphism, conducted exclusively in the female subsample due to the gene’s X-linked localization, also revealed no statistically significant differences between suicidal women and the control group under the additive, dominant, or recessive inheritance models (*p* > 0.05). The *HTR2A rs6313* polymorphism was excluded from interpretation in the female subgroup due to deviation from the Hardy–Weinberg equilibrium in the control group.

Consequently, in women, the *HTR2A rs6311* polymorphism exhibited a consistent and highly significant association with suicide attempts across all inheritance models, while the *SKA2 rs7208505* polymorphism demonstrated a nominally significant sex-specific effect.

## 3. Discussion

In the present study, a robust association was identified between the *rs6311* polymorphism of the *HTR2A* gene and suicide attempts in the Kazakh population, both in the overall sample and after stratification by sex. The CC genotype exhibited a substantial protective effect, while the presence of the T allele, particularly in the homozygous state, was associated with a significantly elevated risk of suicidal behavior. These results align with existing data underscoring the pivotal role of the serotonergic system in modulating affect, impulsivity, and the stress response [95].

The *HTR2A* gene, which is responsible for encoding the 5-HT2A serotonin receptor, is a G protein-coupled receptor that is widely expressed in the prefrontal cortex, hippocampus, and limbic regions. These brain structures are critical for emotional regulation, cognitive control, and behavioral responses to stress [96]. Functionally, the 5-HT2A receptor primarily signals through the Gq/11 protein pathway, activating phospholipase C, increasing intracellular calcium levels, and stimulating protein kinase C-dependent cascades [97]. These processes modulate glutamatergic neurotransmission, synaptic plasticity, and cortical excitability, thereby influencing higher-order cognitive and affective processes.

The *rs6311* polymorphism is located in the *HTR2A* promoter region and is considered functionally significant due to its potential impact on receptor transcriptional activity and expression. The T allele has been demonstrated to be associated with increased promoter activity and higher receptor density in comparison to the C allele [24]. Increased 5-HT2A expression has been demonstrated to enhance cortical excitability and promote hyperreactivity of corticolimbic circuits, potentially lowering the threshold for maladaptive emotional responses and stress sensitivity [98]. Such changes are widely considered to be neurobiological substrates of depression, anxiety disorders, and suicidal behavior [6].

Importantly, 5-HT2A receptors exert a modulatory effect on the balance between inhibitory serotonergic and excitatory glutamatergic signaling [99]. Disruption of this balance has been demonstrated to contribute to impaired emotional regulation, increased impulsivity, and deficient top-down control by the prefrontal cortex. From a neurobiological perspective, *increased 5-HT2A* receptor expression in combination with severe environmental stress may enhance threat processing, impair cognitive flexibility, and weaken top-down regulatory mechanisms mediated by the prefrontal cortex [100]. This model aligns with the stress diathesis concept of suicidal behavior, which posits that genetic predisposition interacts with environmental factors to determine clinical outcomes [101]. Neuroimaging and postmortem studies provide further support for this hypothesis, demonstrating increased *HTR2A* expression and increased receptor density in the prefrontal cortex of individuals who died by suicide [102]. Thus, it can be speculated that carriers of the C allele—especially CC homozygotes, as observed in our study—exhibit relatively reduced receptor expression, which may exert a neuroprotective effect by limiting cortical hyperexcitability and increasing stress resilience [20,103].

An additional regulatory layer influencing *HTR2A* expression includes epigenetic mechanisms. The impact of DNA methylation in the promoter region, in conjunction with glucocorticoid exposure during chronic stress, on transcriptional activity has been demonstrated [104]. Interactions between *rs6311* and environmental factors, including psychosocial stress and traumatic experiences, may further enhance the functional consequences of the T allele [100,105]. It has been proposed that hypomethylation of the promoter region in T allele carriers may serve as a mechanism contributing to stress-induced increases in *HTR2A* expression [23,106,107].

A meta-analysis by Li Sun et al. examining *rs6311* and *rs6313* polymorphisms in patients with schizophrenia did not reveal significant associations with suicidal behavior [108]. These discrepancies may be indicative of variations in diagnostic composition, population structure, or gene–environment interactions, underscoring the intricacy of the genetic underpinnings contributing to suicidality. When interpreting the results of genetic association studies, it is also important to consider population heterogeneity. Allele frequencies, linkage disequilibrium patterns, and gene–environment interactions can vary significantly across ethnic groups, potentially leading to inconsistent results across studies [109].

The clinical significance of *HTR2A* is further substantiated by pharmacological data indicating that 5-HT2A receptor antagonism is one of the mechanisms underlying the therapeutic effects of several serotonergic antidepressants and atypical antipsychotics [110]. Furthermore, mounting evidence indicates that *HTR2A* variability may influence the efficacy of antidepressant treatment, emotional processing, receptor binding affinity, intracellular signaling efficiency, and, consequently, individual variability in treatment efficacy and tolerability [111].

Consequently, *HTR2A* has been proposed as a promising pharmacogenetic marker for predicting therapeutic outcomes and developing personalized treatment strategies for mood disorders [112].

A particularly salient finding was the pronounced effect of *rs6311* observed among the female subjects in this study. This association may be explained by the modulatory effects of sex hormones, particularly estrogens, on serotonergic neurotransmission. Estradiol has been demonstrated to regulate the expression of genes involved in serotonin synthesis (*TPH2*), its degradation (*MAOA*, *MAOB*), transport (*SLC6A4*), and receptor signaling (*HTR1A*) via estrogen receptor-mediated transcriptional mechanisms [113]. In addition to direct DNA binding, estrogen receptors have the capacity to influence gene expression through the action of transcription factors, including *AP-1*, *SP1*, and *NF-κB*, even in the absence of classical estrogen response elements [114].

These mechanisms may partially explain the heightened behavioral impact of *HTR2A* genetic variation in women, particularly under conditions of hormonal fluctuation and stress. This interpretation aligns with the findings of previous studies that have established a link between *rs6311* and depression, anxiety disorders, and suicidality, particularly in female populations and populations exposed to severe stress [115].

The results of this study, when considered collectively, lend support to the hypothesis that the *rs6311* polymorphism in the *HTR2A* gene plays a role in the biological mechanisms underlying suicidal behavior. Furthermore, these results underscore the potential significance of this polymorphism as a molecular marker of suicide risk. In a broader sense, the results underscore the significance of incorporating sex and ethnic disparities into genetic association studies. These findings align with polygenic and gene–environment models that have been developed to elucidate the complex genetics of suicidality. In order to further elucidate the mechanistic role of *HTR2A* in suicidal behavior, there is a necessity for future large-scale studies that will integrate genetic, epigenetic, and neuroimaging data. Despite frequent characterization of the *rs6311* and *rs6313* polymorphisms in the *HTR2A* gene as being in linkage disequilibrium, the magnitude of this association may vary across ethnic groups, attributable to disparities in allele frequencies, recombination patterns, and demographic histories. Consequently, the observed frequency of the minor *rs6311* allele in our cohort is more likely to reflect population-specific genetic architecture than a methodological discrepancy.

Despite the fact that the association between the *rs7208505* polymorphism of the *SKA2* gene and suicide attempts in women was no longer statistically significant after adjustment for multiple testing, its biological basis merits careful consideration. The *SKA2* gene encodes a protein that is part of the spindle-kinetochore-associated complex. In addition to its canonical role in mitotic regulation, the protein is involved in neurobiological processes associated with the nuclear translocation of the glucocorticoid receptor (GR) [65]. This process is critical for cortisol-mediated negative feedback and the timely termination of the stress response [116].

Reduced *SKA2* expression has been associated with impaired GR translocation, decreased cortisol sensitivity, and prolonged HPA axis activation [117]. Prolonged HPA axis activation, in turn, has been associated with elevated cortisol levels, impaired neural plasticity, prefrontal cortex dysfunction, and emotional dysregulation [118,119,120].

The *rs7208505* polymorphism is located in the regulatory region of *SKA2* and has previously been associated with altered gene expression and epigenetic modifications, including promoter DNA methylation [121]. In a recent study, Valenzuela-García et al. (2023) reported that the A allele, consistent with this study’s findings, significantly affects *SKA2* expression levels [122]. The study found that suicide victims exhibited higher expression levels of *SKA2* [122]. Furthermore, Kaminsky et al. (2015) demonstrated that *SKA2* DNA methylation interacts with trauma exposure and predicts suicide attempts with approximately 80% accuracy [123].

In the present study, the observed association was observed only in women, suggesting a potential sex-specific effect. The protective effect associated with the G allele in the recessive model may indicate relatively preserved *SKA2* expression and more efficient glucocorticoid signaling, thereby promoting adaptive regulation of the stress response. Conversely, the AA genotype may hypothetically contribute to impaired HPA axis feedback and increased vulnerability to stress-related psychopathology [122]. Such sex differences may be attributable to hormonally mediated changes in HPA axis regulation, as previously discussed [114].

However, the observed association did not remain statistically significant after adjustment for multiple comparisons. Consequently, these results should be interpreted with caution and considered preliminary. Notwithstanding, the biological plausibility of the *SKA2* gene’s involvement in stress regulation, in conjunction with mounting evidence implicating its genetic and epigenetic variations in suicidal behavior, underscores the necessity for additional large-scale studies that incorporate sex-based distinctions. These studies are imperative to elucidate the potential role of this gene in suicide risk.

In addition to genetic data, some sociodemographic differences were also observed between the groups. Educational level and occupational status varied significantly, with unemployment being more common among patients who had attempted suicide. These results align with the findings of previous studies that have identified socioeconomic vulnerability as a significant correlate of suicidal behavior [124,125]. However, such factors must be regarded as contextual rather than causal, as suicidal behavior is widely recognized as a multifactorial phenotype shaped by a complex interaction between biological predisposition and environmental stressors. It has been demonstrated that socioeconomic adversity can contribute to chronic stress, reduced access to resources, and reduced coping capacity. These factors may interact with underlying genetic risk [126].

These observations further underscore the significance of integrative models that take into account both genetic and psychosocial determinants when studying the etiology of suicidal behavior.

The *FKBP5* gene, which encodes a glucocorticoid receptor co-chaperone and plays a central role in the regulation of the hypothalamic–pituitary–adrenal (HPA) axis, has been associated with stress-related mental disorders and suicidal behavior [56]. In the present study, the *rs3800373* polymorphism did not demonstrate a significant association with suicide attempts. These results are consistent with data indicating that *FKBP5* modulates stress susceptibility primarily through epigenetic regulation of glucocorticoid receptor sensitivity rather than through direct structural and genetic effects [59,61,127,128].

Conversely, polymorphisms in genes associated with serotonin synthesis and metabolism (i.e., *TPH1*, *TPH2*, and *MAOA*) have not been linked to suicide attempts. While these genes do influence monoaminergic tone and behavioral traits such as impulsivity and emotional reactivity, their effects are typically indirect, acting through intermediate phenotypes rather than the suicidal phenotype itself [26,27,28,30,129]. Furthermore, mounting evidence indicates that the impact of *MAOA* variants is predominantly influenced by environmental adversity, including early childhood stress and chronic psychosocial stress, which may obscure direct genetic effects in moderate-sized samples [41,102,130,131]. No significant associations were observed for genes involved in neuroplasticity and dopaminergic signaling (*BDNF*, *COMT*, and *ANKK1*). These genes have been demonstrated to modulate cognitive control, reward processing, and affective regulation. However, the influence of these genes on suicidal behavior appears to be highly context-dependent and is often manifested in the presence of severe psychopathology or chronic stress [132,133].

For instance, although *BDNF* plays a critical role in synaptic plasticity, the *Val66Met* variant has demonstrated inconsistent associations across diverse populations, indicating that it is unlikely to serve as a universal genetic predictor of suicide risk [134]. Similarly, meta-analytic data do not support a direct effect of *COMT rs4680* on suicidal behavior [135,136], while the effect of *ANKK1 rs1800497* may be mediated through intermediate behavioral phenotypes such as impulsivity and reward sensitivity, despite previous reports of an increased risk among TT carriers [50].

The *rs1126647* polymorphism of the *CXCL8* gene demonstrated no association with suicide attempts, suggesting a potential specificity of inflammatory mechanisms in suicidality. Although IL-8 is widely regarded as a pivotal mediator of neuroimmune interactions [137], its regulatory influence is likely to occur primarily through dynamic changes in cytokine expression rather than through stable genetic variants. Decreased IL-8 levels in the cerebrospinal fluid have been reported in individuals with suicidal tendencies [60], as well as possible sex differences in genetic effects [90]. No associations were identified for *rs6355* of the *SLC6A4* gene, likely due to the extremely low frequency of the minor allele and the limited statistical power of the analysis. In contrast to this variant, most studies focus on the functional polymorphism of 5-*HTTLPR* and its interaction with stressors [138]. Concomitantly, the absence of observed associations has been documented in other populations [139].

Recent findings have underscored the significance of intracellular signaling cascades, particularly the cyclic AMP-protein kinase A (PKA) pathway, in the regulation of neuronal plasticity, stress response, and gene transcription processes implicated in suicidal behavior. Dysregulation of PKA-dependent signaling has been associated with affective pathology and may represent an additional molecular layer contributing to suicide vulnerability, complementing genetic variations in the serotonergic and stress response systems [140]. In addition to PKA-dependent mechanisms, postmortem studies have identified dysregulation of protein kinase C isoforms in the prefrontal cortex of individuals with depression and suicidal behavior. This finding suggests that altered intracellular signaling may contribute to impaired synaptic plasticity and affective regulation. These data support the concept that dysregulation of serotonergic receptors may influence broader kinase-mediated signaling pathways involved in suicide vulnerability [141]. In addition to genetic variation, extant evidence suggests that post-translational regulatory mechanisms, including changes in polyamine metabolism and protein modifications such as hypusination, may contribute to the neurobiological architecture of suicidal behavior [142]. Future multi-omics studies integrating genomic and proteomic data may facilitate a more comprehensive understanding of the role of these molecular processes in suicide vulnerability.

Several single-nucleotide polymorphisms (SNPs) were excluded from further analysis due to significant deviation from the Hardy–Weinberg equilibrium or low minor allele frequency. A multitude of factors may give rise to deviations from equilibrium, including but not limited to genotyping errors, population stratification, natural selection, and sampling variability. Given the ethnically homogeneous design of this study and the use of standardized genotyping procedures, technical artifacts are unlikely to fully explain these variations. However, the limited sample size may have resulted in random variation. Consequently, these factors must be taken into account when interpreting genetic results.

The results of this study provide support for the hypothesis that suicidal behavior is influenced by a polygenic and context-dependent architecture. The association of the *HTR2A rs6311* polymorphism, along with the nominal sex-specific signal observed for *SKA2*, underscores the significance of serotonergic receptor regulation and stress axis dysfunction in suicide vulnerability. Conversely, the absence of associations with other candidate genes indicates that their effects are presumably indirect and biologically mediated, underscoring the need for integrative models that encompass genetic, environmental, and sex-specific mechanisms. The merits of this study are manifold. A meticulous case–control design, in which a clinically well-defined phenotypic group of patients who had attempted suicide was utilized, is a notable strength. Additionally, using an ethnically homogeneous sample is a commendable approach that mitigates the risk of population stratification.

A thorough examination of candidate genes, incorporating the serotonergic system, stress response regulation, neuroplasticity, and inflammatory mechanisms, facilitated an integrated analysis of the molecular underpinnings of suicidal behavior. A notable benefit of this approach is the integration of diverse genetic inheritance models and sex stratification, which facilitated the identification of sex-specific associations, particularly for the *SKA2* gene.

However, the study is not without its limitations. Firstly, the relatively modest sample size may limit the statistical power to detect small effects characteristic of polygenic psychiatric phenotypes. Moreover, the analysis concentrated on individual single-nucleotide polymorphisms, omitting an evaluation of haplotypes, gene expression levels, or epigenetic modifications that may play a significant role in the pathogenesis of suicidal behavior. The limited availability of detailed information regarding the impact of environmental factors, including early trauma and chronic stress, impedes the capacity to analyze gene–environment interactions. The study’s findings, based on a sample of the Kazakh population, require validation through further research using larger, more ethnically diverse samples. Correction for multiple comparisons reduced the statistical significance of certain results, underscoring the need for careful interpretation. A notable limitation of this study is the uneven gender distribution in the sample, which precluded a comprehensive gender-specific statistical analysis due to the insufficient sample size of male participants.

### Clinical Implications

The results obtained from this study suggest a potential clinical significance of the *HTR2A rs6311* polymorphism in assessing individual vulnerability to suicidal behavior. Regulatory variants in *HTR2A*, which are associated with altered serotonergic receptor signaling, may facilitate biologically based risk stratification, particularly among individuals with affective dysregulation.

Although the observed association for *SKA2* lost significance after correction for multiple comparisons, its well-established role in regulating the stress axis supports its consideration as a biologically plausible candidate for future research, particularly in the context of sex-specific stress reactivity.

From a translational medicine perspective, the integration of genetic information with clinical and psychosocial data has the potential to enhance multifactorial models of suicide risk assessment and facilitate the development of personalized preventive strategies. Furthermore, genes involved in serotonergic signaling and stress regulation remain promising targets for pharmacogenetic studies aimed at optimizing treatment response. However, the clinical use of genetic markers requires confirmation in larger independent cohorts and should be undertaken with appropriate caution.

## 4. Materials and Methods

### 4.1. Study Design/Sample Size and Groups

The present observational case–control study was conducted to identify genetic and psychosocial factors associated with suicidal behavior in the Kazakh population. The study was conducted at the Astana City Multidisciplinary Hospital in the Republic of Kazakhstan.

The study group consisted of 126 patients who were hospitalized in the toxicology department after attempting suicide by deliberate drug poisoning. The following criteria must be met to be considered for participation in the study: The subject must be at least 18 years of age. The subject must have a confirmed history of self-poisoning by taking medications for suicidal purposes. The subject must be able to understand the information provided and sign a consent form. Individuals with severe cognitive impairment and those who declined to participate were excluded from the study. The comparison group (control group) was formed via simple random sampling and included 120 participants from among university students, faculty members, their partners, and nursing staff at the same institution where the main group was recruited. Participants in the control group were matched with those in the main group on age, gender, and ethnicity. The mental and psychological state of the control group participants was assessed based on an analysis of their medical history, which confirmed the absence of current or previous suicidal intent, attempts, or mental disorders in the participants or their close relatives.

### 4.2. Ethical Considerations

The study was conducted in accordance with the principles of the Declaration of Helsinki and Good Clinical Practice. The study protocol was reviewed and approved by the local ethics committee of L.N. Gumilyov Eurasian National University (Protocol No. 31 dated 11 August 2022). Prior to study inclusion, written informed consent was obtained from all participants.

### 4.3. Genotyping

Biological material for molecular genetic studies (venous blood) was collected from participants in the study and control groups under standardized conditions, adhering to all regulatory requirements and biosafety guidelines. The collection of blood from the cubital vein was performed using sterile 9-mL vacutainers with anticoagulant, in strict accordance with international standards for the collection of biological specimens. For participants in the control group, venous blood was collected on an empty stomach in an outpatient setting using a similar protocol. All samples were promptly transferred to the laboratory at 4 °C and processed within 4 h of collection. Genomic DNA was extracted from 200 μL of whole blood using the commercial QIAamp DNA Blood Mini Kit (Qiagen, Hilden, Germany), following the manufacturer’s instructions. The analysis of the samples revealed that each 200-microliter sample yielded a total DNA yield ranging from 4 to 12 micrograms. The quality and concentration of the DNA were assessed using a spectrophotometric assay (NanoDrop™, Thermo Fisher Scientific, Waltham, MA, USA) at 260/280 nm and by agarose gel electrophoresis. Single-nucleotide polymorphism (SNP) genotyping was performed using the TaqMan^®^ SNP Genotyping Assay (Applied Biosystems, 2017; Thermo Fisher Scientific, USA). The following SNPs were selected for further analysis due to their association with the regulation of serotonergic activity and the risk of suicidal behavior:

- *TPH1* (*rs211105*, *rs7933505*);
- *TPH2* (*rs4570625*, *rs4641528*, *rs13886494*);
- *HTR2A* (*rs6313*, *rs6311*);
- *MAOA* (*rs5906957*);
- *ANKK1* (*rs1800497*);
- *BDNF* (*rs6265*);
- *COMT* (*rs4680*);
- *CXCL8* (*rs1126647*);
- *SKA2* (*rs7208505*);
- *SLC6A4* (*rs6355*);
- *FKBP5* (*rs3800373*).

All reactions were prepared in accordance with the manufacturer’s instructions using standardized reagent concentrations. Thermal cycling conditions included an initial denaturation at 95 °C for 2 min, followed by 40 cycles of denaturation at 95 °C for 10 s and annealing/extension at 56–60 °C. Allelic discrimination was conducted using fluorescently labeled probes with CFX Maestro software (Bio-Rad, Hercules, CA, USA) BIO-RAD CFX384 Real-Time system.

Standard quality-control procedures were applied to ensure the reliability of the genotyping data. Genotyping was performed using automated allele discrimination with manual review of ambiguous results. Approximately 10% of samples were randomly selected for repeat genotyping to ensure 100% concordance.

No samples with excessive missing genotyping data were observed, and the overall genotyping rate exceeded 95% for all analyzed SNPs. Genotyping was performed under standardized laboratory conditions using the same equipment, reagents, and protocols to minimize potential batch effects.

SNPs were excluded from further analysis if they significantly deviated from the Hardy–Weinberg equilibrium in the control group (*p* < 0.05) or exhibited a minor allele frequency below 5%, as such variants have limited statistical power and may reflect technical or population artifacts. Table 1 presents information on 15 single-nucleotide polymorphisms of 11 genes used in the current study.

### 4.4. Statistical Analysis

A statistical analysis of the data was performed using modern biostatistical methods to assess associations between the studied single-nucleotide polymorphisms (SNPs) and the risk of suicide attempt. Prior to the study, the required sample size was estimated using an online sample size calculator (https://www.calculator.net/sample-size-calculator.html, accessed on 10 August 2024) to ensure adequate statistical power. The calculation assumed a 95% confidence level, 95% statistical power, an estimated prevalence of suicidal behavior of 14% in Astana, and a margin of error of 4.77%. The resulting minimum sample size was consistent with the number of participants included in the study, supporting the adequacy of the study design for detecting statistically significant differences between groups.

The Hardy–Weinberg equilibrium (HWE) distribution of genotypes was subsequently examined in the control group using the χ^2^ test. Polymorphisms deviating from HWE (*p* < 0.05), as well as SNPs with a minor allele frequency (MAF) of less than 5%, were excluded from further analysis. The Hardy–Weinberg equilibrium was assessed exclusively in the control group, as is recommended for case–control genetic association studies. Deviations from HWE in the control group were considered indicative of potential genotyping errors, population stratification, or sampling bias. Such variants were excluded from further analysis. Conversely, deviations observed in the case group were not used as exclusion criteria, as they may reflect a true association with the phenotype under study.

The association between genetic variants and suicide attempts was assessed using a logistic regression analysis implemented in the R software environment (R Core Team, version 4.3.0, Vienna, Austria). The analysis was conducted within the framework of additive, dominant, and recessive inheritance models. The regression models incorporated covariates for sex and age. The analysis was further stratified by sex to identify potential sex-specific genetic effects.

The strength of these associations was subsequently assessed through the calculation of odds ratios (ORs) with 95% confidence intervals (95% CI), in addition to the evaluation of χ^2^ test values. The Bonferroni correction was applied to account for multiple comparisons, with the total number of polymorphisms analyzed as the basis for this adjustment. Values of *p* < 0.05 were considered nominally significant, and results that remained significant after correction were interpreted as statistically robust. The Bonferroni correction was applied to account for multiple comparisons, with the number of polymorphisms analyzed (*n* = 15), resulting in an adjusted significance level of *p* < 0.0033.

## 5. Conclusions

In the present study, a significant association was identified between the *rs6311* regulatory polymorphism of the *HTR2A* gene and suicide attempts in the Kazakh population, underscoring the pivotal role of serotonergic receptor signaling in biological vulnerability to suicidal behavior. Despite the robust statistical association found in this study, further clarification is required regarding the biological consequences of the *rs6311* variant. As a regulatory polymorphism located in the promoter region, its functional impact may depend on transcriptional dynamics, epigenetic regulation, and gene–environment interactions. Consequently, there is a necessity for future functional studies that will integrate transcriptomic and epigenetic approaches. These studies will contribute to a more comprehensive understanding of the mechanisms that link *HTR2A* variability to suicidal behavior. A nominal sex-specific association was observed for the *SKA2 rs7208505* variant; however, this association was no longer significant after correction for multiple comparisons. Therefore, the observed result should be interpreted with caution. The absence of associations for several other candidate genes lends support to the notion of a polygenic and context-dependent architecture of suicidal behavior. A comprehensive analysis of the findings reveals an expansion of existing knowledge concerning the molecular underpinnings of suicidality within a population that has received limited study. This expansion underscores the importance of integrative approaches that account for neurobiological, stress-related, and sex-specific mechanisms. Future investigations should incorporate validated psychometric instruments and structured clinical evaluations to enable a more comprehensive characterization of suicidal behavior and to support more precise analyses of genetic associations. Subsequent large-scale studies are required to validate these findings and elucidate the role of regulatory genetic variation in suicide risk.

## Figures and Tables

**Table 1:** Sociodemographic and clinical characteristics of study participants.

Category	Controls (*n* = 120)	Patients (*n* = 126)	*p*-Value
**Age**			0.076 ^1^
mean age ± SD	33 ± 12.74	36.23 ± 15.9	
**Sex**			0.91 ^†^
Women	69.8%	69.2%	
Men	30.2%	30.85%	
**Marital status**			0.07 ^†^
Married	34.5%	44.3%	
Single	61.8%	47.7%	
Divorced/Widowed	3.7%	8%	
**Level of education**			0.01 ^†^
Secondary	51.1%	60.1%	
Incomplete higher education	21.3%	8.6%	
Higher education	27.6%	31.3%	
**Occupation**			<0.001 ^†^
Employed	54.5%	29.2%	
Not employed	8.1%	45.2%	
Student	37.4%	25.6%	
**Clinical Diagnosis**			
F43.2—Adjustment Disorders	85 (67.46%)	-	-
F10—Alcohol-Induced Mental and Behavioral Disorders	17 (13.5%)		
F32.2—Severe Depressive Episode Without Psychotic Symptoms	12 (9.52%)		
Z04.8—Examination and Observation for Other Specified Causes	6 (4.76%)		
F31.1—Bipolar Affective Disorder. Current Episode Manic Without Psychotic Symptoms	3 (2.38%)		
Refusal to Consult a Psychotherapist	3 (2.38%)		

^1^ Continuous variables were compared using Welch’s *t*-test. ^†^ Categorical variables were compared using the chi-square test.

**Table 2:** Overall distribution of genotypes and allele frequencies of the analyzed SNPs in the general study population.

Gene	SNP ^1^	Genotype, *n* (%)	Allele, *n* (%)	χ^2^ (HWE ^2^)	*p*-Value
***TPH1***	*rs211105*	CC—19 (7.72) CT—81 (32.93) TT—146 (59.35)	C—119 (24.19) T—373 (75.81)	2.57	0.277
	*rs7933505*	AA—38 (15.45) AG—166 (67.48) GG—42 (17.07)	A—242 (49.19) G—250 (50.81)	30.46	**<0.001**
***HTR2A***	*rs6313*	AA—33 (13.42) AG—125 (50.81) GG—88 (35.77)	A—191 (38.82) G—301 (61.18)	1.20	0.550
	*rs6311*	CC—59 (23.98) CT—121 (49.19) TT—66 (26.83)	C—239 (48.58) T—253 (51.42)	0.06	0.971
***TPH2***	*rs4570625*	GG—96 (39.03) GT—148 (60.16) TT—2 (0.81)	G—340 (69.11) T—152 (30.89)	774.93	**<0.001**
	*rs4641528*	CC—52 (21.14) CT—121 (49.19) TT—73 (29.67)	C—225 (45.73) T—267 (54.27)	0.02	0.990
	*rs1386494*	TT—49 (19.92) TC—197 (80.08) CC—0 (0.00)	T—295 (60.00) C—197 (40.00)	109.71	**<0.001**
***SLC6A4***	*rs6355*	CC—239 (97.15) CG—7 (2.85) GG—0 (0.00)	C—485 (98.58) G—7 (1.42)	0.05	0.975
***ANKK1***	*rs1800497*	AA—23 (9.35) AG—100 (40.65) GG—123 (50.00)	A—146 (29.67) G—346 (70.33)	0.17	0.920
***BDNF***	*rs6265*	CC—199 (80.89) CT—23 (9.35) TT—24 (9.76)	C—421 (85.57) T—71 (14.43)	95.01	**<0.001**
***COMT***	*rs4680*	AA—39 (15.85) AG—113 (45.94) GG—94 (38.21)	A—191 (38.82) G—301 (61.18)	0.27	0.875
***CXCL8***	*rs1126647*	AT—148 (60.16) AA—89 (36.18) TT—9 (3.66)	A—326 (66.26) T—166 (33.74)	31.72	**<0.001**
***SKA2***	*rs7208505*	AA—70 (28.45) AG—123 (50.00) GG—53 (21.55)	A—263 (53.46) G—229 (46.54)	0.01	0.997
***FKBP5***	*rs3800373*	AA—58 (23.57) AC—129 (52.43) CC—59 (24.00)	A—245 (49.80) C—247 (50.20)	0.586	0.746

^1^ Single-nucleotide polymorphism, ^2^ Hardy–Weinberg equilibrium was assessed in the control group as a quality control measure.

**Table 3:** SNP associations with suicide attempts.

Gene	SNP	Genetic Model	Genotype	Patients (*n* = 126) *n*, %	Controls (*n* = 120) *n*, %	HWE (Control)	OR (95% CI)	Chi-Square	*p*-Value
1	2	3	4	5	6	7	8	9	10
***THP1***	*rs211105*	**additive**	CC	9 (7.14%)	10 (8.33%)	*p* = 0.176 (χ^2^ = 1.8307)	0.85 [0.33–2.16]	0.12707	0.9384
			CT	42 (33.34%)	39 (32.5%)		1.17 [0.69–1.98]		
			TT	75 (59.52%)	71 (59.17%)		1.01 [0.61–1.69]		
		**dominant**	TT	75 (59.52%)	71 (59.17%)		1.01 [0.61–1.69]	0	1
			CT-CC	51 (40.48%)	49 (40.83%)		0.99 [0.59–1.64]		
		**recessive**	TT-CT	117 (92.86%)	110 (91.7%)		1.18 [0.46–3.02]	0.012	0.9118
			CC	9 (7.14%)	10 (8.3%)		0.85 [0.33–2.16]		
***HTR2A***	*rs6313*	**additive**	AA	20 (15.87%)	13 (10.83%)	*p* = 0.0992 (χ^2^ = 2.719)	1.55 [0.73–3.28]	1.7213	0.4229
			AG	60 (47.62%)	65 (54.17%)		0.77 [0.47–1.27]		
			GG	46 (36.51%)	42 (35%)		1.07 [0.63–1.80]		
		**dominant**	GG	46 (36.51%)	42 (35%)		1.07 [0.63–1.80]	0.013	0.9096
			(AA-AG)	80 (63.49%)	78 (65%)		0.94 [0.56–1.58]		
		**recessive**	(AG-GG)	106 (84.13%)	107 (89.17%)		0.64 [0.30–1.36]	0.945	0.331
			AA	20 (15.87%)	13 (10.83%)		1.55 [0.73–3.28]		
***HTR2A***	*rs6311*	**additive**	CC	17 (13.5%)	42 (35%)	*p* = 0.8195 (χ^2^ = 0.0521)	0.29 [0.15–0.55]	22.413	**1.36 × 10^−5^**
			CT	62 (49.2%)	59 (49.17%)		1 [0.61–1.65]		
			TT	47 (37.3%)	19 (15.83%)		3.16 [1.72–5.81]		
		**dominant**	CC	17 (13.5%)	42 (35%)		1.1 [0.66–1.86]	14.438	**0.0001**
			(CT-TT)	109 (86.5%)	78 (65%)		0.29 [0.15–0.55]		
		**recessive**	(CC-CT)	79 (62.7%)	101 (84.17%)		0.32 [0.17–0.58]	13.357	**0.0002**
			TT	47 (37.3%)	19 (15.83%)		3.16 [1.72–5.81]		
***TPH2***	*rs4641528*	**additive**	CC	21 (16.67%)	31 (25.83%)	*p* = 0.5292 (χ^2^ = 0.4669)	0.57 [0.31–1.07]	3.1192	0.2102
			CT	65 (51.58%)	56 (46.67%)		1.22 [0.74–2.01]		
			TT	40 (31.75%)	33 (27.5%)		1.23 [0.71–2.12]		
		**dominant**	TT	40 (31.75%)	33 (27.5%)		1.23 [0.71–2.12]	0.34702	0.5558
			(CT-CC)	86 (68.25%)	87 (72.5%)		0.82 [0.47–1.41]		
		**recessive**	(CT-TT)	105 (83.33%)	89 (74.17%)		1.74 [0.94–3.24]	2.5727	0.1087
			CC	21 (16.67%)	31 (25.83%)		0.57 [0.31–1.07]		
***ANKK1***	*rs1800497*	**additive**	AA	11 (8.73%)	12 (10%)	*p* = 0.6985 (χ^2^ = 0.15)	0.86 [0.36–2.03]	0.14047	0.9322
			AG	51 (40.47%)	49 (40.83%)		0.99 [0.59–1.64]		
			GG	64 (50.8%)	59 (49.17%)		1.07 [0.65–1.76]		
		**dominant**	GG	64 (50.8%)	59 (49.17%)		1.07 [0.65–1.76]	0.016	0.8985
			AA-AG	62 (49.2%)	61 (50.83%)		0.94 [0.57–1.54]		
		**recessive**	AG-GG	115 (91.27%)	108 (10%)		1.16 [0.49–2.74]	0.015	0.9022
			AA	11 (8.73%)	12 (90%)		0.86 [0.36–2.03]		
***COMT***	*rs4680*	**additive**	AA	25 (19.84%)	14 (11.67%)	*p* = 0.887 (χ^2^ = 0.0202)	1.87 [0.92–3.81]	3.7189	0.1558
			AG	58 (46.03%)	55 (45.83%)		1.01 [0.61–1.66]		
			GG	43 (34.13%)	51 (42.5%)		0.7 [0.42–1.17]		
		**dominant**	GG	43 (34.13%)	51 (42.5%)		0.7 [0.42–1.17]	1.4877	0.2226
			(AA-AG)	83 (65.87%)	69 (57.5%)		1.43 [0.85–2.39]		
		**recessive**	(AG-GG)	101 (80.16%)	106 (88.33%)		0.53 [0.26–1.08]	2.4965	0.1141
			AA	25 (19.84%)	14 (11.67%)		1.87 [0.92–3.81]		
***SKA2***	*rs7208505*	**additive**	AA	41 (32.54%)	29 (24.17%)	*p* = 0.715 (χ^2^ = 0.1333)	1.51 [0.86–2.65]	2.3921	0.3024
			AG	61 (48.41%)	62 (51.66%)		0.88 [0.53–1.45]		
			GG	24 (19.04%)	29 (24.17%)		0.74 [0.4–1.36]		
		**dominant**	GG	24 (19.04%)	29 (24.17%)		0.74 [0.4–1.36]	0.67408	0.4116
			(AA-AG)	102 (80.96%)	91 (75.83%)		1.35 [0.74–2.49]		
		**recessive**	(AG-GG)	41 (32.54%)	29 (24.17%)		0.66 [0.38–1.16]	1.7253	0.189
			AA	85 (67.46%)	91 (75.83%)		1.51 [0.86–2.65]		
***FKBP5***	*rs3800373*	**additive**	AA	25 (19.84%)	33 (27.5%)	*p* = 0.4168 (χ^2^ = 0.6594)	1.04 [0.56–1.96]	3.8315	0.1472
			AC	65 (51.59%)	64 (53.33%)		0.93 [0.57–1.54]		
			CC	36 (28.57%)	23 (19.17%)		1.69 [0.93–3.06]		
		**dominant**	CC	36 (28.57%)	23 (19.17%)		1.69 [0.93–3.06]	2.4883	0.1147
			(AA-AC)	90 (71.43%)	97 (80.83%)		0.59 [0.33–1.08]		
		**recessive**	AA	25 (19.84%)	33 (27.5%)		1.04 [0.56–1.96]	1.5984	0.2061
			(AC-CC)	101 (80.16%)	87 (72.5%)		1.53 [0.85–2.77]		

**Table 4:** SNP associations with suicide attempts among women.

Gene	SNP	Genetic Model	Genotype	Patients (*n* = 88) *n*, %	Controls (*n* = 83) *n*, %	HWE (Control)	OR (95% CI)	Chi-Square	*p*-Value
1	2	3	4	5	6		7	8	9
***THP1***	*rs211105*	**additive**	CC	8 (9.09%)	7 (8.43%)	*p* = 0.253 (χ^2^ = 1.3065)	1.09 [0.38. 3.14]	0.0885	0.9567
			CT	30 (34.09%)	27 (32.53%)		1.02 [0.54–1.93]		
			TT	50 (56.82%)	49 (59.04%)		0.91 [0.5–1.68]		
		**dominant**	TT	50 (56.82)	49 (59.04%)		0.91 [0.5–1.68]	0.0192	0.8897
			CT-CC	38 (43.12%)	34 (40.96)		1.55 [0.81–2.96]		
		**recessive**	TT-CT	80 (90.91%)	76 (91.57%)		0.92 [0.32–2.66]	1.0317 × 10^−29^	1
			CC	8 (9.09%)	7 (8.43%)		1.09 [0.38–3.14]		
***HTR2A***	*rs6311*	**additive**	CC	13 (14.77%)	31 (37.35%)	*p* = 0.2971 (χ^2^ = 1.087)	0.29 [0.14–0.61]	20.137	**4.24 × 10^−5^**
			CT	43 (48.87%)	43 (51.81%)		0.89 [0.49–1.62]		
			TT	32 (36.36%)	9 (10.84%)		4.70 [2.08–10.64]		
		**dominant**	CC	13 (14.77%)	31 (37.35%)		0.29 [0.14–0.61]	37.722	**8.16 × 10^−10^**
			(CT-TT)	75 (85.23%)	52 (62.65%)		3.44 [1.64–7.19]		
		**recessive**	(CC-CT)	56 (63.64%)	74 (89.16%)		0.21 [0.09–0.48]	13.894	**0.0001**
			TT	32 (36.36%)	9 (10.84%)		4.70 [2.08–10.64]		
***TPH2***	*rs4641528*	**additive**	CC	13(14.77%)	21 (25.3%)	*p* = 0.4488 (χ^2^ = 0.5737)	0.51 [0.24–1.1]	2.9994	0.2232
			CT	47 (53.41)	38 (45.78%)		1.36 [0.74–2.48]		
			TT	28 (31.82%)	24 (28.92%)		1.15 [0.6–2.2]		
		**dominant**	TT	28 (31.82%)	24 (28.92%)		1.15 [0.6–2.2]	0.0605	0.8056
			(CT-CC)	60 (68.18%)	59 (71.08%)		0.87 [0.45–1.67]		
		**recessive**	(CT-TT)	75 (85.23%)	62 (74.7%)		1.95 [0.91–4.22]	2.3481	0.1254
			CC	13 (14.77%)	21 (25.3%)		0.51 [0.24–1.1]		
***ANKK1***	*rs1800497*	**additive**	AA	8 (9.1%)	10 (12.05%)	*p* = 0.1442 (χ^2^ = 2.1328)	0.73 [0.27–1.95]	1.0581	0.5892
			AG	37 (42.04%)	29 (34.94%)		1.35 [0.73–2.51]		
			GG	43 (48.86%)	44 (53.01%)		0.85 [0.46–1.54]		
		**dominant**	GG	43 (48.86%)	44 (53.01%)		0.85 [0.46–1.54]	0.1515	0.6971
			AA-AG	45 (51.14%)	39 (45.99%)		1.18 [0.65–2.15]		
		**recessive**	AG-GG	80 (90.9)	73 (87.95%)		1.37 [0.51–3.66]	0.1447	0.7036
			AA	8 (9.1%)	10 (12.05%)		0.73 [0.27–1.95]		
***COMT***	*rs4680*	**additive**	AA	15 (17.04%)	9 (10.84%)	*p* = 0.7019 (χ^2^ = 0.1465)	1.69 [0.7–4.1]	0.5394	0.4631
			AG	41 (46.6%)	39 (46.99%)		0.98 [0.54–1.8]		
			GG	32 (36.36%)	35 (42.17%)		0.78 [0.42–1.45]		
		**dominant**	GG	32 (36.36%)	35 (42.17%)		0.78 [0.42–1.45]	0.3849	0.5349
			(AA-AG)	56 (63.64%)	48 (57.83%)		1.28 [0.69–2.36]		
		**recessive**	(AG-GG)	73 (82.96%)	74 (89.16%)		0.59 [0.24–1.44]	0.8962	0.3438
			AA	15 (17.04%)	9 (10.84%)		1.69 [0.7–4.1]		
***SKA2***	*rs7208505*	**additive**	AA	30 (34.1%)	15 (18.07%)	*p* = 0.0946 (χ^2^ = 2.7939)	2.34 [1.15–4.78]	5.7959	**0.05**
			AG	40 (45.45%)	49 (59.04%)		0.58 [0.32–1.06]		
			GG	18 (20.45%)	19 (22.89%)		0.87 [0.42–1.79]		
		**dominant**	GG	18 (20.45%)	20 (24.1%)		0.87 [0.42–1.79]	0.1509	0.6977
			(AA-AG)	70 (79.55%)	63 (75.9%)		1.15 [0.56–2.39]		
		**recessive**	(AG-GG)	58 (65.9%)	68 (81.93%)		0.43 [0.21–0.87]	4.4864	**0.0275**
			AA	30 (34.1%)	15 (18.07%)		2.34 [1.15–4.78]		
***FKBP5***	*rs3800373*	**additive**	AA	16 (18.18%)	20 (24.1%)	*p* = 0.1428 (χ^2^ = 2.1471)	0.7 [0.33–1.47]	1.9945	0.3689
			AC	49 (55.68%)	48 (57.83%)		0.92 [0.5–1.68]		
			CC	23 (26.14%)	15 (18.07%)		1.6 [0.77–3.34]		
		**dominant**	CC	23 (26.14%)	15 (18.07%)		1.6 [0.77–3.34]	1.1744	0.2785
			(AA-AC)	65 (73.86%)	68 (81.93%)		0.62 [0.3–1.3]		
		**recessive**	(AC-CC)	72 (81.82%)	63 (75.9%)		1.43 [0.68–2.99]	0.5783	0.447
			AA	16 (18.18%)	20 (24.1%)		0.7 [0.33–1.47]		
***MAOA***	*rs5906957*	**additive**	AA	22 (25%)	17 (20.49%)	*p* = 0.8221 (χ^2^ = 0.0505)	1.29 [0.63–2.66]	2.4525	0.2934
			AG	32 (36.36%)	40 (48.19%)		0.61 [0.33–1.13]		
			GG	34 (38.64%)	26 (31.32%)		1.38 [0.73–2.6]		
		**dominant**	GG	34 (38.64%)	26 (31.32%)		1.38 [0.73–2.6]	0.7	0.4
			(AA-AG)	54 (61.36%)	57 (68.68%)		0.72 [0.39–1.36]		
		**recessive**	(AG-GG)	66 (75%)	66 (79.52%)		0.77 [0.38–1.59]	0.2718	0.6021
			AA	22 (25%)	17 (20.48%)		1.29 [0.63–2.66]		

**Table 5:** Characteristics of single-nucleotide polymorphisms included in the study.

Graph.	Single-Nucleotide Polymorphism	Locus	Additional Functions and Associations
*TPH1* (*tryptophan hydroxylase 1*)	*rs211105*	11p15.3	Rate-limiting enzyme of serotonin synthesis [13]. Variations can affect serotonin levels in the central nervous system, which is linked to depression, anxiety, and impulsive behavior [12]. It is associated with seasonal affective disorder, aggression, and suicidality [37].
	*rs7933505*		
*HTR2A* (*5-hydroxytryptamine receptor 2A*)	*rs6313*	7q21.22	Involved in the transmission of serotonin signals [10]. It is associated with sensory processing, learning, memory, and mental disorders such as schizophrenia and bipolar disorder [74,75]. It influences neuronal sensitivity to serotonin, which may modulate the effects of antidepressants [76].
	*rs6311*		
*MAOA* (*monoamine oxidase*)	*rs5906957*	Xq13.1	An enzyme that breaks down monoamines, including serotonin. Variations are associated with aggressive behavior, antisocial personality traits, and addiction [36,77,78].
*TPH2* (*tryptophan hydroxylase 2*)	*rs4570625*	12p13.31	Rate-limiting enzyme of serotonin synthesis [14]. It influences serotonin levels in peripheral tissues and may be linked to metabolic disorders [79]. It is associated with depression, anxiety, and seasonal affective disorder [80].
	*rs4641528*		
	*rs13886494*		
*SLC6A4* (*serotonin transporter*)	*rs6355*	17q11.2	Encodes the serotonin transporter, which is involved in the regulation of serotonin levels in synapses [32]. Associated with depression, anxiety disorders, impulsive behavior, and suicidality [34,81].
*ANKK1* (*ankyrin repeat and kinase domain containing 1*)	*rs1800497*	11q23.2	Associated with the dopamine D2 receptor, it is involved in dopaminergic transmission [49]. Associated with nervous system development and cytoskeletal formation [47]. Associated with various neurological and psychiatric disorders, including schizophrenia and bipolar disorder [82].
*BDNF* (*brain-derived neurotrophic factor*)	*rs6265*	11p14.1	It encodes the BDNF protein, which is important for the growth, differentiation, and survival of neurons [83]. Regulates neuronal survival, growth, and differentiation [58]. Associated with depression, anxiety disorders, cognitive function, and neurodegenerative diseases [68,84,85].
*COMT* (*catechol-O-methyltransferase*)	*rs4680*	22q11.21	Encodes the enzyme catechol-O-methyltransferase, which is involved in the metabolism of catecholamines (dopamine, epinephrine, serotonin, and norepinephrine) [52]. The *Val158Met* variant affects dopamine regulation in the prefrontal cortex and is associated with cognitive function, stress, and depressive disorders [86,87,88,89].
*CXCL8* (*C-X-C motif chemokine ligand 8*)	*rs1126647*	4q13-q21	Encodes the cytokine IL-8, which attracts neutrophils to the site of inflammation [90]. Associated with inflammatory processes, as well as mental illnesses such as depression and schizophrenia [90,91,92].
*SKA2* (*spindle and kinetochore associated complex subunit 2*)	*rs7208505*	17q21.31	Involved in the regulation of mitosis and the control of cell proliferation [63]. Regulates the activity of other proteins through phosphorylation. Associated with various diseases, including cancer and neurodegenerative disorders [64].
*FKBP5* (*FKBP prolyl isomerase 5*)	*rs3800373*	6q15	A molecular chaperone involved in the folding of glucocorticoid receptors (GCRs). GCRs play an important role in regulating the body’s stress response [93]. Polymorphisms of the *FKBP5* gene, including rs3800373, are associated with increased sensitivity to stress and an increased risk of developing post-traumatic stress disorder (PTSD) [94]. Genetic variations in *FKBP5* may influence the efficiency of the stress system, making individuals more vulnerable to developing mental disorders, including depression and suicidal ideation [57].

## Data Availability

The original contributions presented in this study are included in the article/Supplementary Materials. Further inquiries can be directed to the corresponding authors.

## Supplementary Materials

The following supporting information can be downloaded at: https://www.mdpi.com/article/10.3390/ijms27052294/s1.
