# Supplementary material for: A Study of Candidate Genes Associated with Suicide Attempts in the Kazakh Population in Astana, Kazakhstan
Source: Int J Mol Sci. 2026 Feb 28;27(5):2294. doi: 10.3390/ijms27052294 (PMC12984681; doi:10.3390/ijms27052294)
Supplement: Supplementary file 1 [file ijms-27-02294-s001.zip › ijms-4138340-supplementary.pdf]

Supplementary Table S1. Allele frequencies of analyzed SNPs in the total sample, controls, and patients.

Gene	SNP[1]	Allele	Total		Controls		Patients	
			n=492	AF[2]	n=240	AF	n=252	AF
THP1	*rs211105*	C	119	0,24	59	0,24	60	0,25
		T	373	0,76	181	0,76	192	0,75
THP1	*rs7933505*	A	242	0,5	121	0,5	121	0,5
		G	250	0,5	119	0,5	131	0,5
HTR2A	*rs6313*	A	191	0,39	91	0,38	100	0,62
		G	301	0,61	149	0,62	152	0,38
HTR2A	*rs6311*	C	239	0,48	143	0,6	96	0,38
		T	253	0,52	97	0,4	156	0,62
TPH2	*rs4570625*	G	340	0,7	161	0,64	183	0,72
		T	152	0,3	79	0,36	59	0,28
TPH2	*rs4641528*	C	225	0,46	118	0,5	107	0,42
		T	267	0,54	122	0,5	145	0,58
TPH2	*rs1386494*	T	295	0,6	143	0,6	152	0,6
		C	197	0,4	97	0,4	100	0,4
SLC6A4	*rs6355*	C	485	0,99	234	0,975	251	0,99
		G	7	0,1	6	0,025	1	0,1
ANKK1	*rs1800497*	A	146	0,3	73	0,3	73	0,29
		G	346	0,7	167	0,7	179	0,71
BDNF	*rs6265*	C	421	0,85	206	0,85	37	0,15
		T	71	0,15	34	0,15	215	0,85
COMT	*rs4680*	A	191	0,39	83	0,34	108	0,42
		G	301	0,61	157	0,66	144	0,58
CXCL8	*rs1126647*	A	326	0,67	120	0,5	192	0,76
		T	166	0,33	120	0,5	60	0,24
SKA2	*rs7208505*	A	263	0,53	120	0,5	143	0,57
		G	229	0,47	120	0,5	109	0,43
SLC6A4	*rs6355*	C	485	0,99	234	0,975	251	0,99
		G	7	0,1	6	0,025	1	0,1
FKBP5	*rs3800373*	A	245	0,5	130	0,54	115	0,46
		C	247	0,5	110	0,16	137	0,54

[1]Single-nucleotide polymorphism, [2]Allele frequency
